# In Vivo Evaluation of the Anti-Inflammatory Activity of Electrospun Micro/Nanofibrous Patches Loaded with *Pinus halepensis* Bark Extract on Hairless Mice Skin

**DOI:** 10.3390/ma12162596

**Published:** 2019-08-15

**Authors:** Eleftheria Kotroni, Eleftheria Simirioti, Stefanos Kikionis, Ioannis Sfiniadakis, Aggeliki Siamidi, Vangelis Karalis, Andreas Vitsos, Marilena Vlachou, Efstathia Ioannou, Vassilios Roussis, Michail Rallis

**Affiliations:** 1 Section of Pharmaceutical Technology, Department of Pharmacy, School of Health Sciences, National and Kapodistrian University of Athens, Athens 15784, Greece; 2 Section of Pharmacognosy and Chemistry of Natural Products, Department of Pharmacy, School of Health Sciences, National and Kapodistrian University of Athens, Athens 15771, Greece; 3 Athens Naval Hospital, Pathologoanatomic Laboratory, Athens 11521, Greece

**Keywords:** *Pinus halepensis* bark extract, skin, UV-induced inflammation, mice, electrospun nanofibrous patches, anti-inflammatory activity

## Abstract

Skin inflammation is the most common symptom in dermatological diseases. It is usually treated by topically applied products, such as creams, gels and lotions. Skin dressings offer a promising alternative as they are endowed with more controlled administration conditions. In this study, the anti-inflammatory activity of electrospun alginate micro/nanofibrous dressings loaded with the aqueous extract of *Pinus halepensis* bark (PHBE) was evaluated in vivo in mice. The upper back skin of SKH-1 female hairless mice was exposed to a single dose of ultraviolet radiation (3 MEDs) and the inflamed area was treated daily by the direct application of a nanofibrous patch. The condition of the skin was evaluated primarily on the basis of clinical observation, photo-documentation and histopathological assessment, while measurements of the erythema, hydration, transepidermal water loss (TEWL) and sebum production were also taken into account. The results showed that the topical application of alginate micro/nanofibrous dressings loaded with PHBE on UV-inflamed skin significantly attenuated inflammation damage, reducing the healing period. Increase of the loading dose of PHBE resulted in a proportional reduction of the extent, the density and the depth of skin inflammation. With the steadily increasing interest of the skin dressing industry towards nanofibrous matrices, electrospun nonwovens could serve as ideal candidates for the development of multifunctional anti-inflammatory care systems.

## 1. Introduction

Skin inflammation is the most common symptom observed in almost all dermatological diseases [1]. Exposure to UV irradiation causes acute and chronic effects on skin, such as inflammation, aging and cancer [2]. The UV-induced reaction depends on the intensity of solar radiation and the time of exposure [3,4], with acute exposure leading to skin damage, such as edema, erythema, dry skin and itching [5,6]. UV radiation generates reactive oxygen species (ROS) resulting in oxidative stress and increase of inflammatory mediators [7,8]. Compounds with radical scavenging properties, e.g., polyphenols, prevent or attenuate the adverse effects of UV radiation on the skin [4,9].

Currently, inflamed skin is usually treated by topically applied products, such as creams, gels and lotions, with their main disadvantage being the non-controlled application. Topical patches seem to be promising as they are endowed with controlled administration properties, possessing the ability to continuously stay in contact with inflamed skin. In addition, they protect the skin surrounding the inflamed area, thus preventing and controlling microbial growth [10,11].

Topical patches can be prepared from a variety of synthetic or natural polymers. Among them, alginate dressings, consisting of alginic acid salts extracted from brown seaweeds, find extensive use in wound care treatment, due to their ability to promote and maintain a physiologically moist environment. As calcium salts, alginates are very effective in heavily exuding wounds; however, they release calcium ions at the wound site, stimulating the production of pro-inflammatory cytokines. This activity, although beneficial in many wound treatments, is not encouraged for the suppression of local inflammation. Hence, sodium alginate dressings are preferred for enhancement of the healing process in dry wounds, where hydrating properties are sought and reduction of the inflammation response is needed [12].

Recently, nanotechnology has opened new possibilities in the fabrication of multifunctional patches. Nanofibrous patches have attracted interest due to their unique properties allowing for a wide range of applications in the biomedical sector, such as drug release, wound healing and tissue engineering systems [13,14,15,16,17,18,19,20]. Under the application of a high voltage electric field, micro/nanofibers can be generated through an electrically charged polymer solution [21,22,23]. A variety of active agents can be incorporated in electrospun fibers of various biocompatible and biodegradable, natural or synthetic polymers, for the fabrication of multifunctional fibrous patches [24,25,26]. Possessing high encapsulation efficacy, high surface-to-volume area, high and controlled porosity, mechanical flexibility and architectural analogy to natural extracellular matrix, electrospun patches loaded with an appropriate active ingredient can serve as ideal candidates for the development of topical anti-inflammatory dressings.

Species of the genus *Pinus* are widely known for their medicinal properties associated with their chemical composition. In many studies, their essential oils and extracts from bark, needles or cones have exhibited antioxidant, antiviral, analgesic, cytotoxic, anti-inflammatory and/or antimicrobial activities [27] and have been utilized for various therapeutic applications [28]. *Pinus halepensis* Miller (Aleppo pine) is among the most commonly growing conifer species in the Mediterranean area. According to previous studies, the aqueous extract of *P*. *halepensis* bark (PHBE), rich in antioxidant agents (polyphenols) [29], including mainly procyanidins and phenolic acids [30], significantly decreases and/or prevents the damage caused by UV radiation or X-ray irradiation to the skin [3,4,30,31,32].

In this study, sodium alginate electrospun micro/nanofibrous patches loaded with PHBE were prepared and used as dressings on the UV-inflamed skin of SKH-1 female hairless mice. Nanofibrous patches were characterized by scanning electron microscopy (SEM) analysis, IR spectroscopy and wettability measurements. Clinical evaluation, photo-documentation and histopathological analyses of the skin were performed and parameters, such as erythema, hydration, transepidermal water loss (TEWL) and sebum production, were assessed and statistically analyzed.

## 2. Materials and Methods

### 2.1. Materials

Cellulose acetate (CA) (MW ~50,000) and polyethylene oxide (PEO) (MW 8,000,000) were purchased from Sigma-Aldrich (Darmstadt, Germany). Sodium alginate (SA) (MW 216,121) was purchased from Cellco Chemicals SA (Athens, Greece). *Pinus halepensis* bark was collected from Kaisariani forest, a suburb near Athens, Greece. A voucher specimen has been deposited at the Herbarium of the Section of Pharmacognosy and Chemistry of Natural Products, Department of Pharmacy, National and Kapodistrian University of Athens (ATPH/TP0115). The bark was pulverized in a blender and extracted with dH_2_O in a 1:10 ratio for 48 h at 40 °C. The extract was filtered and freeze-dried to afford a solid residue. All reagents used were of analytical grade. Sterile gauze (Asepta Gauze^®^, Asepta, Athens, Greece) and adhesive tapes (Aseptafix^®^, Asepta, Athens, Greece; Rolltex^®^ Skin, Master Aid, Capannori, Italy; Tegaderm^TM^, 3M, St. Paul, MN, USA) were purchased from a local pharmacy store (Athens, Greece).

### 2.2. Preparation of the Micro/Nanofibrous Patches by Electrospinning

Spinning solutions were prepared by dissolving the polymers in the appropriate solvent system. Specifically, CA/PHBE spinning solutions were prepared by dissolving CA at 9% w/v in a solvent system of acetone/H_2_O (9:1 v/v) followed by the addition of PHBE at four different concentrations of 0.5%, 1%, 2% and 4% w/v. For the PEO/SA solutions, PEO at 1.5% w/v and SA at 3% w/v were dissolved in H_2_O. Neat solutions of 9% w/v CA in acetone/H_2_O (9:1 v/v) and 4.5% w/v PEO in H_2_O were prepared. All spinning solutions were prepared at room temperature under stirring for 24 h to ensure their homogeneity.

Electrospinning was conducted using a *γ*-High Voltage Research DC power supply generator (Gamma High Voltage Research, Ormond Beach, FL, USA) with a maximum voltage of 50 kV. The polymer solutions were loaded into 10-mL disposable syringes fitted with 23G tip-ground-to-flat needles. To ensure the homogeneity of the blended fiber mats, electrospinning was performed on an antiparallel setup with the syringes mounted on two Harvard PHD 2000 programmable syringe pumps (Harvard Apparatus, Holliston, MA, USA). The produced nanofibers were deposited on aluminum foil wrapped on a RC-6000 (NaBond Technologies, Hong Kong) rotating drum collector at a rotation speed of 400 rpm. Temperature and relative humidity were 20 ± 2 °C and 60 ± 5%, respectively. Applied voltage was fixed at 25 kV. Tip-to-collector distance was fixed at 10 cm for the CA and CA/PHBE solutions, and at 20 cm for the PEO and PEO/SA solutions.

The spinning solutions were electrospun in different combinations to afford different blended fibrous matrices, namely CA-PEO, CA-(PEO/SA), (CA/PHBE)-(PEO/SA)-A, (CA/PHBE)-(PEO/SA)-B, (CA/PHBE)-(PEO/SA)-C and (CA/PHBE)-(PEO/SA)-D. Specifically, the CA-PEO fiber mats were fabricated from the electrospinning of CA and PEO spinning solutions, while the CA-(PEO/SA) fiber mats were fabricated from the CA and PEO/SA solutions. Finally, the (CA/PHBE)-(PEO/SA)-A, (CA/PHBE)-(PEO/SA)-B, (CA/PHBE)-(PEO/SA)-C and (CA/PHBE)-(PEO/SA)-D fiber mats were fabricated from the CA/PHBE solutions at four different concentrations of PHBE (0.5%, 1%, 2% and 4% w/v, respectively) electrospun with the PEO/SA solution. CA and CA/PHBE solution feeding rates were fixed at 1 mL/h, with PEO and PEO/SA solution feeding rates adjusted at 0.5 mL/h, resulting to a 2:1 (w/w) blending ratio of CA or CA/PHBE fibers to PEO or PEO/SA fibers, respectively, in the fabricated matrices. The exact composition of the blended electrospun micro/nanofibrous mats of different weight ratios is shown in Table 1.

### 2.3. Characterization of the Micro/Nanofibrous Patches

Scanning electron microscopy: A PhenomWorld desktop scanning electron microscope (SEM; Phenom-World, Eindhoven, The Netherlands) with tungsten filament (10 kV) and charge reduction sample holder was used for the morphological characterization of the nanofibers. The diameters of 100 fibers from each SEM image were measured using the embedded image analysis software (Phenom Pro Suite/Fibermetric, Eindhoven, The Netherlands) and the average fiber diameter was determined.

IR spectroscopy: The chemical composition of the fibers was analyzed by Fourier transform infrared spectroscopy using the attenuated total reflection (ATR) method on a FTIR Bruker Alpha II spectrometer (Bruker Optics Inc., Billerica, MA, USA).

Water uptake studies: The water uptake ability of the fabricated patches was evaluated by immersion of pre-weighted lyophilized samples (1% w/v) in dH_2_O at RT for different time intervals (30 sec, 1 h, 4 h and 24 h). After each time interval the excess surface water was poured and removed by placing the wet sample on a blotting paper for 1 min. Subsequently, the wet sample was weighted and the degree of water uptake was calculated by the following equation:

$$Water\;Uptake\;\left(\%\right)=\;\frac{W_{w}-W_{d}}{W_{d}}\times 100$$

where *W_w_* is the weight of the wet patch and *W_d_* is the initial weight of the dry patch. Measurements were performed in triplicate.

### 2.4. Animals and Study Design

All procedures performed were carried out in accordance with the guidelines established by the European Communities Council Directive (Directive 2010/63/EU of 22 September 2010). The experimental procedure was approved by the National Peripheral Veterinary Authority Animal Ethics Committee (Protocol Numbers: 3255/7-06-2017 and 3278/27-06-2018) after the affirmative opinion of the Animal Protocols Evaluation Committee. 

Female hairless mice, type SKH-1, three-nine months old were used for this study. All mice originated from the breeding stock of the Department of Pharmacy Small Animal Laboratory (EL 25 BIO 07). The animal room temperature and humidity were maintained at 24 ± 1 °C and 40 ± 10%, respectively. The room was illuminated under a 12 h cycle of light and dark. The mice had unrestricted continuous access to solid pellets (Nuevo SA, N. Artaki, Greece and Farma Efyra Industrial & Commercial SA, Corinth, Greece) and fresh water.

The mice were divided into seven groups (each consisted of six to eight mice): 1) mice exposed to UV light and treated with sterile gauze; 2) mice exposed to UV light and treated with CA-PEO patches; 3) mice exposed to UV light and treated with CA-(PEO/SA) patches; 4) mice exposed to UV light and treated with (CA/PHBE)-(PEO/SA)-A patches; 5) mice exposed to UV light and treated with (CA/PHBE)-(PEO/SA)-B patches; 6) mice exposed UV light and treated with (CA/PHBE)-(PEO/SA)-C patches; and 7) mice exposed UV light and treated with (CA/PHBE)-(PEO/SA)-D patches. After UV irradiation, the damaged area was treated daily by placing electrospun patches of equal size covered using a combination of different types of adhesive tapes (Aseptafix^®^, Rolltex^®^ Skin or Tegaderm^TM^) in order to prevent the mice from removing the dressing.

### 2.5. UV-Induced Inflammation

Solar-simulated UV radiation (290–400 nm) was emitted from an Oriel 1000 W Xenon lamp on a 66021 Universal Arc Lamp Housing (Oriel Instruments, Stratford, CT, USA) equipped with appropriate wavelengths filters and powered by a 68820 Universal Power Supply (Oriel Instruments, Stratford, CT, USA). The irradiation dose was measured before every experiment by Oriel Goldilux Radiometer/Photometer UVB and UVA probes (Oriel Instruments, Stratford, CT, USA). The irradiation was adjusted at 10 mW/cm^2^ for UVA and at 14.3 mW/cm^2^ for UVB. A 4 cm^2^ area of the upper part of the animals’ back was once exposed to 3 Minimal Erythemal UV light Doses (MEDs). The total irradiation dose that each mouse received was 150 mJ/cm^2^ for the UVA and 214.5 mJ/cm^2^ for UVB.

### 2.6. Clinical Evaluation, Photo-Documentation and Histopathological Analysis

The clinical condition of mice (e.g., skin inflammation intensity and mobility) was recorded daily. The animals were weighted at day 1, 4, 7 and 10. Skin images for photo-documentation were acquired using a Nikon D5100 digital camera (Nikon, Tokyo, Japan) equipped with a AF-S Micro Nikkor 60 mm f/2.8 G ED lens (Nikon, Tokyo, Japan), which was at a fixed distance of 33 cm perpendicular to the subject.

At the end of the experiment, which lasted for 10 days when no inflammation could be observed for at least one group of treated mice, the mice were sacrificed and skin tissue was excised. Skin sections of specimens from all groups were performed using a paraffin microtome (Shandon Finesse, Thermo Scientific, USA) and stained with haematoxylin & eosin stain kit (Atom Scientific, Cheshire, UK). Parameters such as inflammation, hyperkeratosis, parakeratosis and skin structure, were estimated.

### 2.7. Evaluation of Skin Parameters

Skin parameters, including erythema, skin hydration, TEWL and sebum production, were evaluated with non-invasive biophysical methods on days 1 (before UV irradiation), 4, 7 and 10. Erythema was calculated by absorption/reflection of three different light wavelengths using a Mexameter MX 18 (Courage + Khazaka electronic GmbH, Köln, Germany). Skin hydration was measured through changes in the dielectric constant using a Corneometer CM 820 (Courage + Khazaka electronic GmbH, Köln, Germany). The water barrier function of skin (TEWL) was evaluated by measuring the density gradient of the water evaporation from the skin using a Tewameter TM 210 (Courage + Khazaka electronic GmbH, Köln, Germany), with the estimation being based on the mean value of the flux density of water (in g/m^2^/h) which was obtained 1 min after the beginning of the measurement. Sebum produced was measured with a Sebumeter SM 810 (Courage + Khazaka electronic GmbH, Köln, Germany) estimating the refractive index of the tape sampling the skin surface lipids. Before each measurement, the treated area was cleaned by wiping the skin surface with sterile gauze. For erythema and hydration, the corresponding probes were applied and the indications were recorded in arbitrary units.

### 2.8. Data Analysis

The results were expressed as mean values ± SD. Statistical differences were estimated using analysis of variance (ANOVA) single factor test and were considered significant when *p* < 0.05.

## 3. Results and Discussion

### 3.1. Physicochemical Characterization of Electrospun Patches

In the present study, alginate micro/nanofibrous patches loaded with PHBE as the active agent were prepared by electrospinning and evaluated for their anti-inflammatory activity. Sodium alginate (SA) is widely used in wound dressings as an antibacterial agent. Capable of absorbing fluids, due to its gelling ability on the treated surface, it can maintain the moisture of the wound environment at physiological levels and promote the skin recovery process. Nevertheless, in lack of good electrospinnability, it is usually electrospun in blends with other polymers [33,34]. To overcome this drawback, polyethylene oxide (PEO), an easily electrospinnable non-toxic polymer, was selected in our study as the polymer carrier for the fabrication of the alginate fibrous non-woven patches. Furthermore, with its hydrophilic and high absorbent character, PEO can facilitate the diffusion of PHBE from the fibers by increasing the wettability of the designed patches, thus potentially enhancing their healing properties.

An initial attempt to electrospin PHBE from the PEO/SA solution was proven unsuccessful, since PHBE was not readily soluble in the PEO/SA aqueous solution, resulting in the formation of aggregates. Therefore, cellulose acetate (CA), a biopolymer of choice in the fabrication of electrospun composite fibers, membranes and films in many drug delivery and other biomedical applications [35,36], was selected as a polymer carrier for PHBE. Considering its relative hydrophobicity and its mechanical properties, CA could also provide the desired mechanical stability in the designed patches.

**Figure 1:**
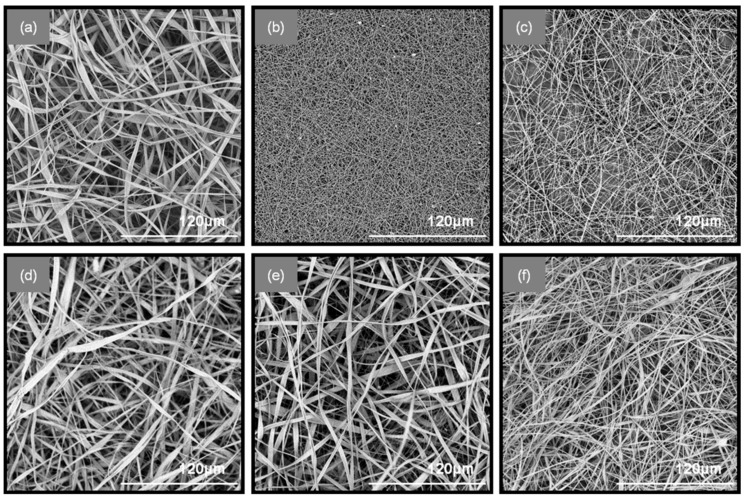
SEM images of (**a**) CA, (**b**) PEO, (**c**) PEO/SA, (**d**) CA-PEO, (**e**) CA-(PEO/SA) and (**f**) (CA/PHBE)-(PEO/SA)-D patches.

The successful blending of the fibers in the patches was achieved through an antiparallel electrospinning setup, in which the two spinning solutions (PEO/SA and CA/PHBE) were electrospun simultaneously on the same rotating drum collector. Specifically, six types of fibrous patches composed of CA-PEO, CA-(PEO/SA) and (CA/PHBE)-(PEO/SA) containing PHBE in four different concentrations were fabricated for the needs of the in vivo evaluation of their anti-inflammatory activity. The electrospinning parameters were fine-tuned in order to obtain uniform, bead-free fibrous mats, as evidenced from the images obtained using Scanning Electron Microscopy (SEM) (Figure 1). When CA and PEO are electrospun from neat solutions, ribbon-like fibers of an average diameter of 2.3 ± 0.54 μm and cylindrical fibers of 210 ± 12 nm average diameter size are observed for CA and PEO fibers, respectively. When PEO/SA blends were electrospun, fibers of similar cylindrical morphology but increased diameter size compared to the PEO fibers, measured at 360 ± 72 nm, were produced. In the blended fibrous patches containing both CA and PEO, mixed ribbon-like and cylindrical fiber morphologies were observed for all co-electrospun matrices. In the CA-PEO fiber mat, the average diameter size was measured at 2.2 ± 0.54 μm, while in the CA-(PEO/SA) fiber mat, the average diameter was measured at 2.0 ± 0.44 μm. A decrease in the fiber size was observed in the blended (CA/PHBE)-(PEO/SA) patches due to the incorporation of the PHBE in the CA fibers. The different amounts of PHBE loaded into the fibers did not affect significantly the morphology or the size of the fibers among the four (CA/PHBE)-(PEO/SA) patches. Specifically, the average fiber diameter was measured at 1.1 ± 0.3 μm for the (CA/PHBE)-(PEO/SA)-A, 1.2 ± 0.27 μm for the (CA/PHBE)-(PEO/SA)-B, 1.1 ± 0.24 μm for the (CA/PHBE)-(PEO/SA)-C and 0.9 ± 0.22 μm for the (CA/PHBE)-(PEO/SA)-D.

**Figure 2:**
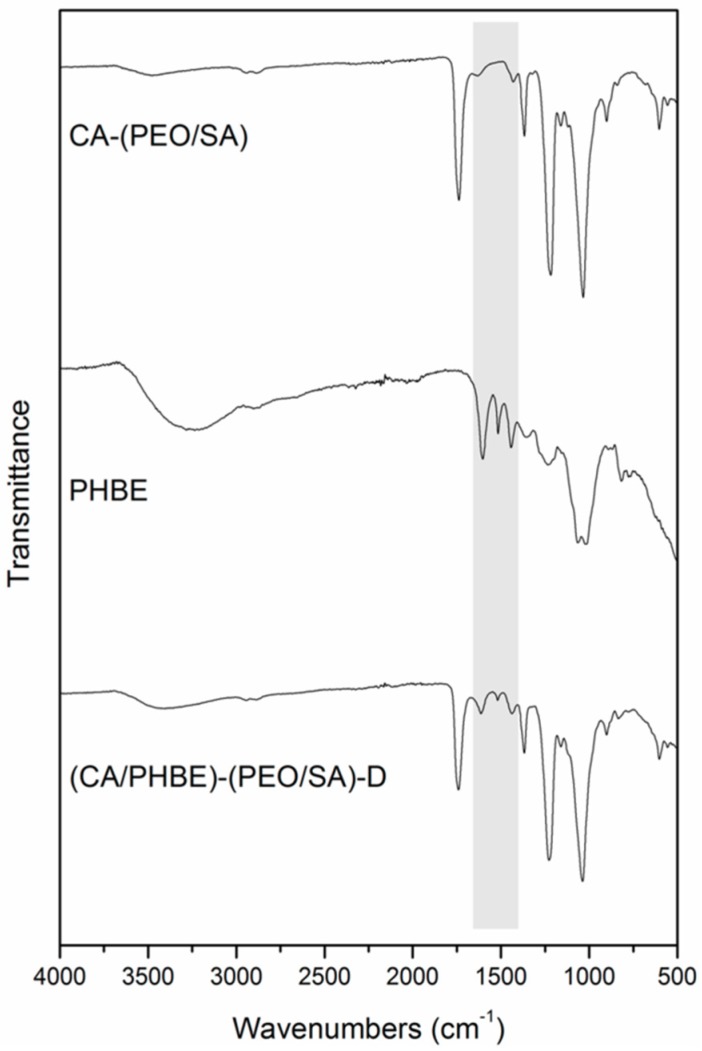
FT-IR spectra of CA-(PEO/SA), PHBE and (CA/PHBE)-(PEO/SA)-D patches.

FT-IR spectroscopic analysis of the fabricated patches revealed the successful incorporation of PHBE in the fiber mats (Figure 2). In the IR spectrum of the (CA/PHBE)-(PEO/SA)-D fiber mat, the absorption band at 1740 cm^−1^ due to the –C=O stretching vibration of CA-(PEO/SA), together with the absorption bands at 1613, 1518 and 1443 cm^−1^ assigned to the aromatic –C=C stretching vibration of PHBE, confirmed the presence of PHBE in the mat. The PHBE loading was further supported from the gradual shift of the characteristic bands of the carrier components. The two absorption bands at 1037 and 1226 cm^−1^ assigned to the –C–O–C stretching vibration revealed a shift to higher wavelengths, as compared to 1033 and 1218 cm^−1^, respectively, in CA-(PEO/SA), while the –OH stretching vibration at 3403 cm^−1^ was shifted to different wavelengths, as compared to 3486 and 3255 cm^−1^ in CA-(PEO/SA) and PHBE, respectively.

Water uptake ability measurements showed that the fabricated patches were capable of uptaking and absorbing water immediately after their immersion in water. All fabricated patches demonstrated similar wettability that was independent of the different loading amount of PHBE. At a time interval of 30 s the water uptake was measured at 174%, while after the first hour the water absorption reached 595%. After 4 h and 24 h, the immersed samples did not show significant increase in their water absorption, with their water uptake values measured at 626% and 634%, respectively, indicating that they reached their water uptake maximum almost within the first hour. This high wettability of the fabricated patches is of high importance for their wound healing function, allowing for the maintenance of a moist wound environment and the diffusion of the embedded pine bark extract.

### 3.2. Evaluation of Skin Parameters

The in vivo anti-inflammatory activity of sodium alginate electrospun micro/nanofibrous patches loaded with PHBE were evaluated as dressings on the UV-inflamed skin of SKH-1 female hairless mice. SKH-1 mice were selected since they are the most widely used in dermatologic research, especially for the evaluation of topical agents [37].

The upper back skin of SKH-1 female hairless mice was exposed to a single dose of ultraviolet radiation (3 MEDs) and the inflamed area was treated daily by the direct application of a nanofibrous patch. The condition of the skin was evaluated primarily on the basis of daily clinical observation, as well as photo-documentation and histopathological assessment at the end of the experiment which lasted for 10 days, while measurements of the erythema, hydration, TEWL and sebum production were also taken into account.

The values of erythema, hydration, TEWL and sebum production are closely related to the degree of inflammation. Specifically, when the inflammation is at a maximum, erythema and TEWL increase, whereas hydration and sebum production decrease.

**Figure 3:**
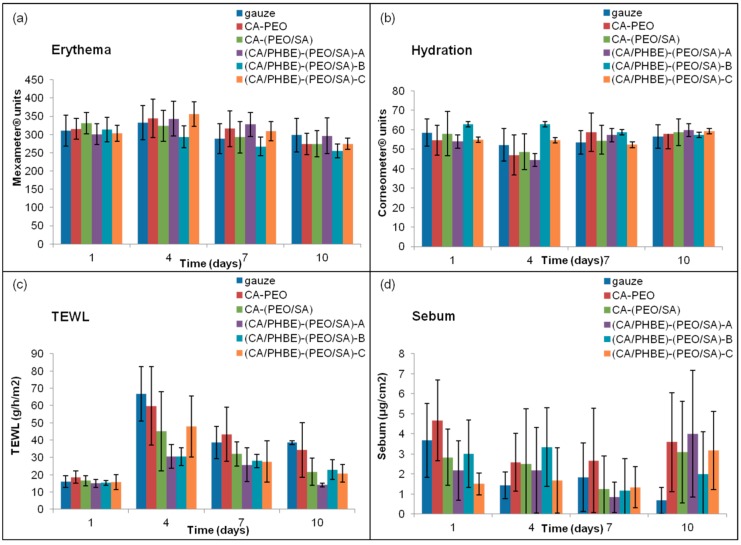
Histograms of (**a**) erythema, (**b**) hydration, (**c**) TEWL and (**d**) sebum production in relation to time after UV exposure.

The patterns of erythema, hydration, TEWL and sebum production of the mice groups after UV irradiation are presented in Figure 3. The highest erythema values were observed on day 4 and returned to the initial levels on day 10 (Figure 3a). On day 4, the time of the maximum inflammation, the groups treated with patches containing the higher doses of PHBE were able to keep the hydration levels of stratum corneum to normal values (Figure 3b). Mice treated with CA-PEO, CA-(PEO/SA) and (CA/PHBE)-(PEO/SA)-A patches showed hydration values lower than those at the beginning (day 1) or the end (day 10) of the experiment (*p* < 0.05). TEWL measurements were realized in order to evaluate the skin barrier function, especially the state of the outer layer of the skin (stratum corneum) during the treatment of UV inflamed skin. On day 1, before UV exposure, the stratum corneum is intact, the barrier function is normal and so TEWL values are minimal. After inflammation with UV radiation, the stratum corneum structure changes and TEWL values become maximal [38]. In all cases TEWL increased significantly (*p* < 0.05) on day 4 and continued to be higher in all cases up to day 7 (*p* < 0.05) (Figure 3c). TEWL was significantly higher than normal in the control (gauze) and CA-PEO groups (*p* < 0.05) at all time points after UV exposure. Patches loaded with PHBE were able to re-establish normal skin barrier and enable stratum corneum to return to its physiological state. In contrast, the sebum production values did not exhibit statistically significant variation (Figure 3d), with the exception of the control (gauze) group where a gradual but significant (*p* < 0.05) decrease of sebum production was observed.

### 3.3. Clinical Evaluation, Photo-Documentation and Histopathological Analysis

Even though measurements of the erythema, hydration, TEWL and sebum production can be considered indicative parameters of the skin condition, the most important diagnostic criteria for the successful treatment of inflammation include photo-documentation and histopathological assessment.

**Figure 4:**
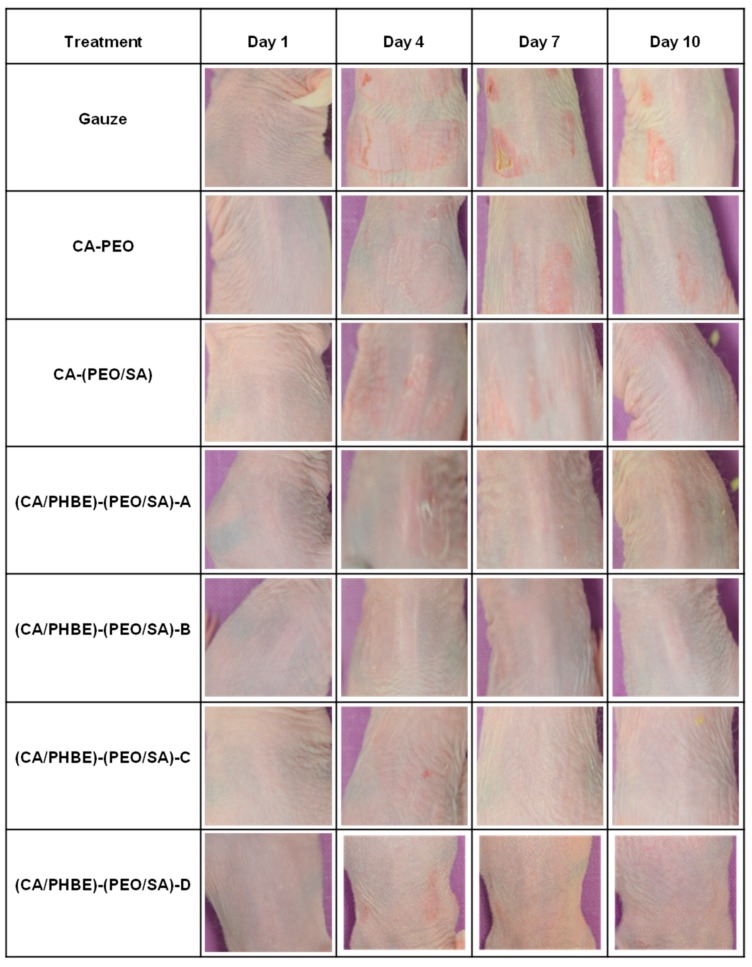
Representative images of the mice back skin before (day 1), during (days 4 and 7) and at the end (day 10) of the treatment period.

In the present study, the clinical evaluation of skin inflammation, in accordance with photo-documentation (Figure 4), showed that patches containing PHBE demonstrated significant anti-inflammatory efficacy in a dose-dependent manner.

**Figure 5:**
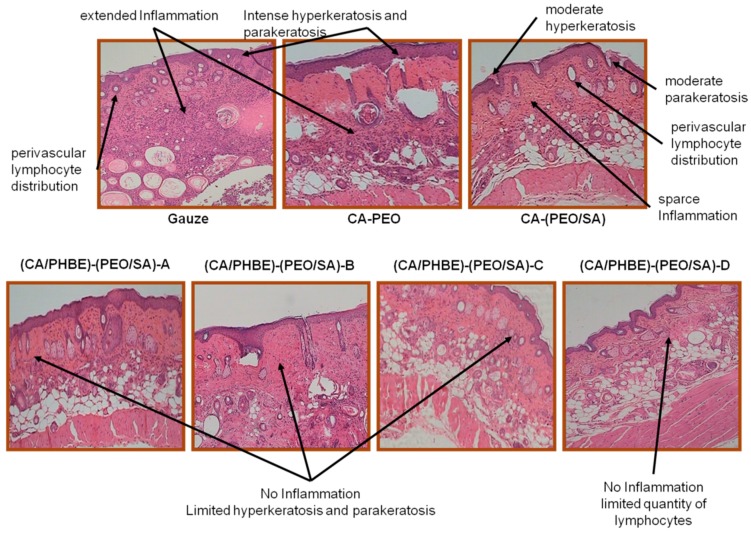
Histological evaluation: skin sections of the different mice groups (magnification 100×).

Histopathological observations of representative skin biopsies are depicted in Figure 5. The skin, especially in dermis and epidermis, of control mice using gauze showed extended inflammation with perivascular lymphocyte distribution, intense hyperkeratosis and parakeratosis; while in control mice treated with CA-PEO patches intense hyperkeratosis was observed as well. In the skin of the control mice using the CA-(PEO/SA) patches, apart from perivascular lymphocyte distribution, moderate hyperkeratosis and parakeratosis and sparse inflammation were observed, probably due to the mild anti-inflammatory properties of SA and its contribution to the skin recovery process. The (CA/PHBE)-(PEO/SA)-A, (CA/PHBE)-(PEO/SA)-B, (CA/PHBE)-(PEO/SA)-C and (CA/PHBE)-(PEO/SA)-D mice groups showed a dose-dependent reduction of skin inflammation. There was no inflammation in the dermis, limited hyperkeratosis and parakeratosis. In particular, the skin of the (CA/PHBE)-(PEO/SA)-D mice group did not show any hyperkeratosis, but only a very limited quantity of lymphocytes (non-inflammatory state). Therefore, it can be safely assumed that PHBE significantly soothes inflamed skin and treats hyperkeratosis. The above results are in accordance with previous observations regarding the protective effect of *P. halepensis* bark extract on the skin [4].

## 4. Conclusions

In summary, the anti-inflammatory activity of electrospun alginate micro/nanofibrous patches loaded with the bark extract of *P. halepensis* was evaluated in vivo in SKH-1 female hairless mice. The topical application of the produced PHBE-loaded micro/nanofibrous dressings, after UV radiation-induced inflammation, ameliorated the damage caused to the skin. By increasing the loading dose of PHBE, a reduction on the extent, density and depth of inflammation in the skin was observed, while the presence of SA as a component of the carrier polymer seems to enhance the anti-inflammatory activity of the designed patches. All patches loaded with the *P. halepensis* bark extract showed statistically significant anti-inflammatory efficacy compared to the controls, with the patch containing 26.2% w/w PHBE (which is the highest concentration that can be successfully incorporated and electrospun) showing the best anti-inflammatory activity. Considering their controlled administration properties and their tailor-made characteristics, electrospun micro/nanofibrous dressings, loaded with various anti-inflammatory agents, could serve as the next generation of anti-inflammatory care systems in skin dressing technology. With the steadily increasing interest of the skin dressing industry towards nanofibrous matrices, electrospun nonwovens could serve as ideal candidates for the development of multifunctional anti-inflammatory care systems, which at the moment are not commercially available for the treatment of UV-inflamed skin, or to a step further for the treatment of side-effects to the skin after radiation therapy of cancer patients.

## Figures and Tables

**Table 1:** Electrospun micro/nanofibrous mats composed of different weight ratios of pine bark extract.

	% w/w/patch			
Patch	CA ^1^	PHBE ^2^	PEO ^3^	SA ^4^
CA-PEO	80	0	20	0
CA-(PEO/SA)	80	0	6.7	13.3
(CA/PHBE)-(PEO/SA)-A	76.6	4.3	6.4	12.8
(CA/PHBE)-(PEO/SA)-B	73.5	8.2	6.1	12.2
(CA/PHBE)-(PEO/SA)-C	67.9	15.1	5.7	11.3
(CA/PHBE)-(PEO/SA)-D	59.0	26.2	4.9	9.8

^1^ Cellulose acetate; ^2^ *Pinus halepensis* bark aqueous extract; ^3^ Polyethylene oxide; ^4^ Sodium alginate.
